# Newtonian boreal forest ecology: The Scots pine ecosystem as an example

**DOI:** 10.1371/journal.pone.0177927

**Published:** 2017-06-14

**Authors:** Pertti Hari, Tuomas Aakala, Juho Aalto, Jaana Bäck, Jaakko Hollmén, Kalev Jõgiste, Kourosh Kabiri Koupaei, Mika A. Kähkönen, Mikko Korpela, Liisa Kulmala, Eero Nikinmaa, Jukka Pumpanen, Mirja Salkinoja-Salonen, Pauliina Schiestl-Aalto, Asko Simojoki, Mikko Havimo

**Affiliations:** 1 Department of Forest Sciences, University of Helsinki, FI University of Helsinki, Finland; 2 Hyytiälä Forestry Field Station, Hyytiäläntie 124, Korkeakoski, Finland; 3 Department of Information and Computer Science, Aalto University School of Science, FI Aalto, Finland; 4 Department of Forest Biology, Estonian University of Life Sciences, Friedrich Reinhold Kreutzwaldi 1, Tartu, Estonia; 5 Department of Food and Environmental Sciences, University of Helsinki, FI University Of Helsinki, Finland; 6 Department of Environmental and Biological Sciences, University of Eastern Finland, FI Kuopio, Finland; Ecole Pratique des Hautes Etudes, FRANCE

## Abstract

Isaac Newton's approach to developing theories in his book Principia Mathematica proceeds in four steps. First, he defines various concepts, second, he formulates axioms utilising the concepts, third, he mathematically analyses the behaviour of the system defined by the concepts and axioms obtaining predictions and fourth, he tests the predictions with measurements. In this study, we formulated our theory of boreal forest ecosystems, called NewtonForest, following the four steps introduced by Newton. The forest ecosystem is a complicated entity and hence we needed altogether 27 concepts to describe the material and energy flows in the metabolism of trees, ground vegetation and microbes in the soil, and to describe the regularities in tree structure. Thirtyfour axioms described the most important features in the behaviour of the forest ecosystem. We utilised numerical simulations in the analysis of the behaviour of the system resulting in clear predictions that could be tested with field data. We collected retrospective time series of diameters and heights for test material from 6 stands in southern Finland and five stands in Estonia. The numerical simulations succeeded to predict the measured diameters and heights, providing clear corroboration with our theory.

## Introduction

The development of a Scots pine dominated forest ecosystem is highly regular following a stand-replacing disturbance. Initially, ground vegetation dominates the ecosystem. Tree seedlings start growing slowly and eventually overcome the ground vegetation.

Forest ecological and physiological research has resulted in valuable results on the metabolism and growth of trees and ground vegetation and of the decomposition of organic matter in the soil, to a large extent thanks to the development of measurement techniques such as chamber techniques and subsequent advances in eddy covariance measurements. The analysis of measurements has resulted in important knowledge on the effects of the environment on gas exchange [1], the structural relationships between tree organs [2] and the active role of the soil microbes in nutrient recycling [3,4]. Permanent sample plots and other growth and yield studies together with forest inventories have provided important information concerning stand growth and development [5–7].

Physics was facing a similar situation, as forest ecology now, in the late 17^th^ century, when theoretical explanations for the observations by Tyko Brahe and Johannes Kepler, and Galileo Galilei’s experiments were missing. Isaac Newton studied the movements of planets around the sun and he explained the orbiting of planets with gravitation.

**Fig 1:**
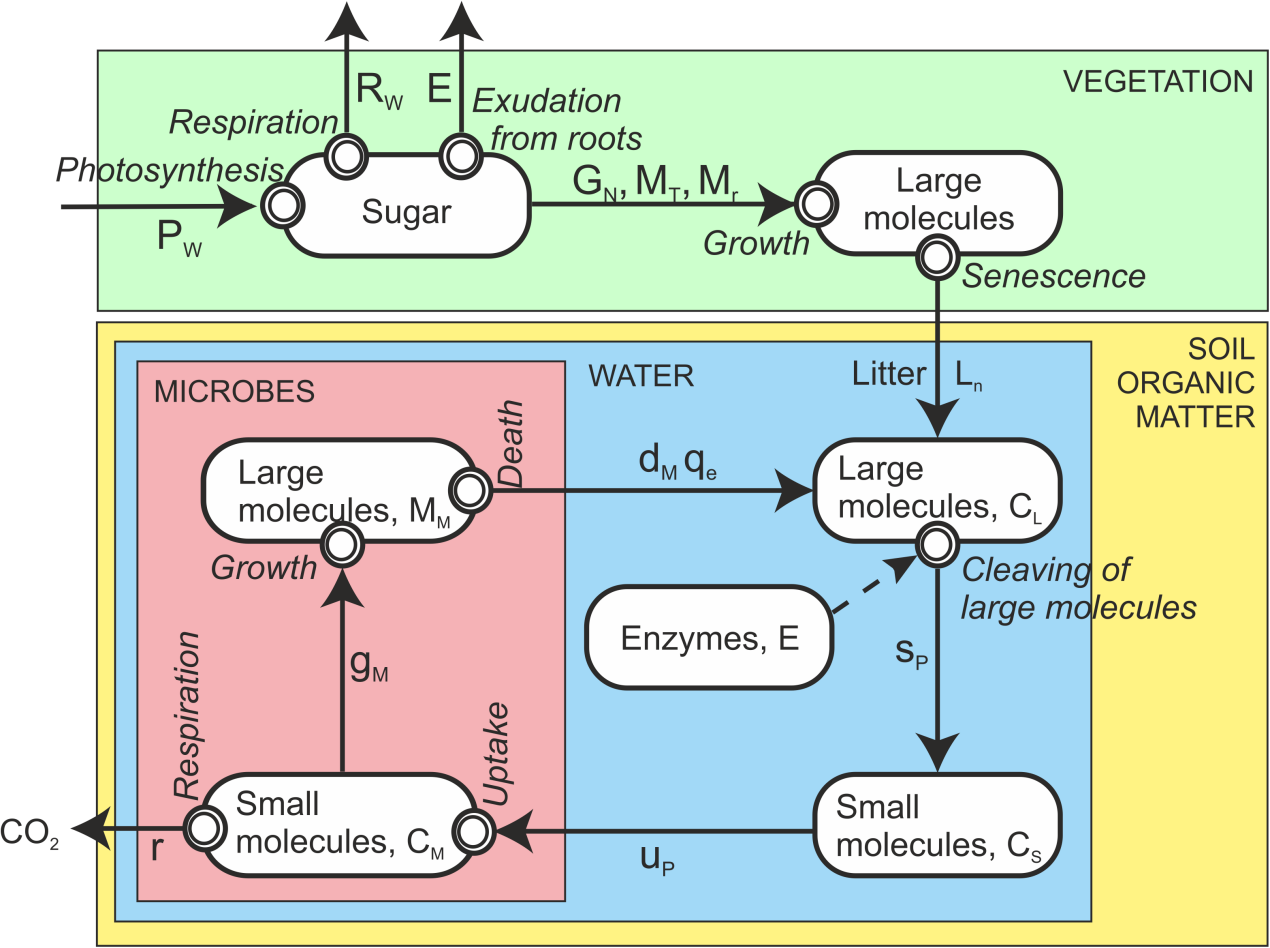
The fluxes of carbon compounds in the forest ecosystem from the atmosphere via leaves and from microbes back to the atmosphere. Boxes indicate amounts, arrows indicate flows, and double rings conversion of carbon compounds.

**Fig 2:**
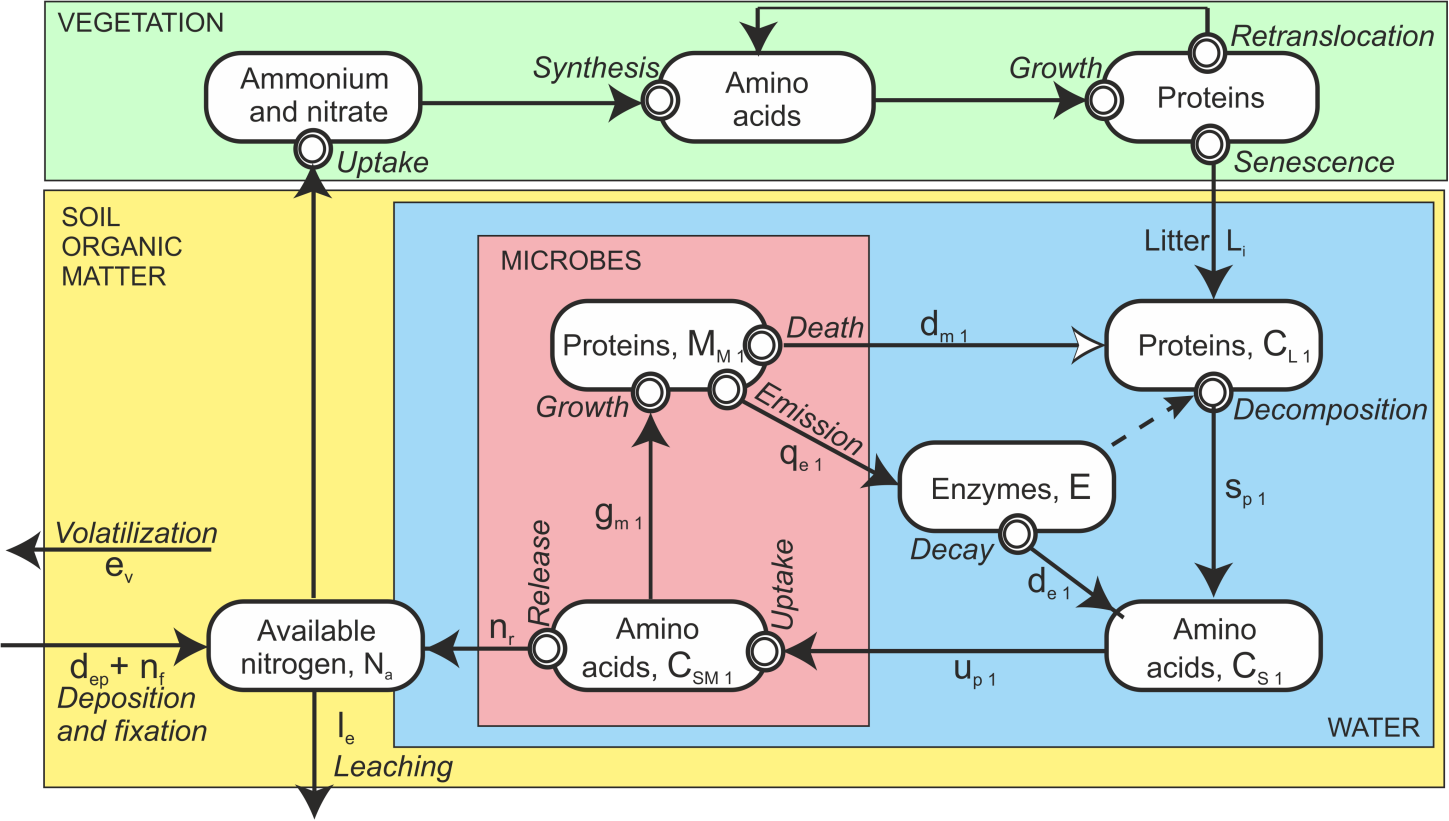
The circulation of nitrogen compounds in the forest ecosystem from available nitrogen in the soil into the metabolism of trees and microbes back to available nitrogen. Boxes indicate amounts, arrows flows, and double rings conversion of nitrogen compounds.

In forest ecosystem metabolism of vegetation and microbes consumes and releases material, and at the same time physical phenomena convert energy to other forms resulting in concentration, pressure and temperature differences. These differences give rise to material and energy flows within atmosphere, vegetation and soil and between atmosphere, vegetation and soil. We hypothesize that these flows accumulate and consume material in vegetation and in soil. On a molecular level, large carbon molecules, i.e., cellulose, lignin, lipids, starch and proteins, form the structure of living systems. Proteins, that are nitrogen-rich molecules, play a key role in the metabolism as e.g. enzymes and membrane pumps [8–10]. We study the flows of these carbon- and nitrogen-rich molecules and processes generating the flows (Figs 1 and 2).

The normal practise in the development of models is that the model structures are derived with quite loose hypothesis and assumptions. In our methodology, we split the phenomenon under study into steps. We define exactly the concept needed for verbal characterisation of the steps. We use axioms utilising the defined concepts to introduce exact description of the regularities in each step, first in verbal form and later with equations. Thus, axioms are close to normal hypothesis and assumptions in modelling, but they are more demanding and exact. Finally, we combine the steps in the mathematical analysis and in this way we derive the dynamic model of forest ecosystem, called NewtonForest. We hypothesize that processes generate carbon and nitrogen flows that give rise to the development of forest ecosystem, and we obtain predictions dealing with the behaviour of Scots pine ecosystem. To test the hypothesis, we studied the flows of carbon and nitrogen compounds and processes generating the flows.

Inspired by Newton's thinking presented in *Principia Mathematica*, we set out to use the same approach also in forest ecology. Our aim is to develop a theory for the boreal forest ecosystem, and to discover the regularities in the metabolism of trees, ground vegetation and microbes and in the structure of trees that explain the regular development of boreal forest ecosystems. We focus on the interaction between trees and soil and on development of trees of different sizes. We use the Scots pine ecosystem as an example.

## Definitions and axioms to characterize a forest ecosystem

### Time scales

The metabolism of trees, ground vegetation and microbes react to the prevailing environment. In contrast, the structures of trees and ground vegetation have clear annual properties. Trees have a very powerful regulation system that determines the annual properties of the structure. Although the growth is reacting to the prevailing environment, the regulation system determines the annual properties of the structure and we have to study tree structure in the annual time scale. When we study the properties of tree structure, we have to combine the metabolism and the regulation of the tree structure. We solve the problem of two time scales by determining the amounts of metabolites produced during a year and feed these amounts into the annual analysis of tree structure.

The metabolism of trees, ground vegetation and microbes in the soil generates changes in the structures of trees and ground vegetation and in the material fluxes and pools in forest ecosystems during the rotation period. We formulate our theory using Scots pine (*Pinus sylvestris* L.) forest as an example. We use simultaneously immediate and annual timescales. We begin our Newtonian analysis of forest ecosystem by defining the concepts and axioms that describe the metabolism that generate the annual changes in the structure of trees and ground vegetation as well as in the material and microbial pools in the soil. Thereafter, we introduce the long-term development of forest ecosystems by piling the changes on each other in the mathematical analysis with numeric simulation methods.

### Trees

#### Metabolism

In the formation of new structures, i.e. in the growth, new cells are formed in shoots, branches, stem, transport roots and fine roots. Sugars and amino acids are the raw materials for growth. Enzymatic reactions synthesise cellulose and lignin for cell walls, lipids for membranes and proteins for enzymes, membrane pumps and pigment complexes, and starch for raw material pools. These syntheses require energy that is obtained via respiration from sugars. Photosynthesis, which is highly variable on temporal and spatial scales, provides the energy and raw material for growth in the form of sugars.

Plant metabolism in the boreal forest has a conspicuous annual cycle, and it is most active during the growing season. Very high metabolic activity develops in late spring and early summer.

The environmental factors affect the metabolism of trees, which results in a close connection between metabolism and the environment. The reactions of metabolism environmental factors are fast. The concentrations and activities of enzymes, membrane pumps and pigments change during the growing season and consequently the relationship between the environment and metabolism changes slowly over prolonged periods. Scots pine trees grow buds with needle embryos in late summer for the next growing season. The structure of the annual rings provides perhaps the most well-known and clearest evidence of the annual time scale of the formation of tree structure.

The structure of forest canopy is very inhomogeneous. Problems with great temporal and spatial variation have been widely analysed in physics. In these problems, the mathematical concept of the density of process rate or of flux is derived from theoretical concepts dealing with processes and material flows. We utilise the physical knowledge and introduce theoretical and mathematical concepts to deal with the problem of great variation.

*Definition 1. Photosynthesis in a point of space and time is the ratio of the amount of sugars produced in a small space element during a short time interval to the product of the needle mass in the volume and the length of the time interval*.

Exact mathematical quantification of photosynthesis in a point in space and time results in the density of photosynthetic rate. This kind of mathematical formalism is commonly used in the physics of inhomogeneous materials [11].

The changes of the concentrations and activities of enzymes, membrane pumps and pigments are characteristic of tree metabolism.

*Definition 2. The regulation system of metabolism controls the synthesis and decomposition of the enzymes, membrane pumps and pigment complexes*.

*Definition 3. The action of the regulation system of metabolism generates the states of the enzymes, membrane pumps and pigments*.

As a result of changing environmental factors, the state of the enzymes, membrane pumps and pigments is under constant slow change, and thus the relationship between photosynthesis and light and temperature changes gradually [11,12].

**Axiom 1. Light, temperature, and the state of the enzymes, pigments and membrane pumps determine photosynthesis in a point of space and time**.

*Definition 4. The annual photosynthetic production in a point is the ratio of the amount of sugars produced in a small volume during a year to the amount of needles in the volume*.

Mathematics provides the connection between photosynthesis and photosynthetic production.

**Axiom 2. The density of photosynthesis in a point of space and time determines the density of annual photosynthetic production**.

We can express axiom 2 more precisely with mathematical notations. Let p denote the density of photosynthetic rate, P_k_ the density of annual photosynthetic production during the year k and t time in point x in the canopy. The axiom 2 results with mathematical notations

$$P_{k}(x)=\int_{t_{k}}^{t_{k+1}}p(t,\;x)\;dt$$ (1)

where *t*_*k*_ is the beginning moment of the year *k*.

According to axiom 1, the density of photosynthetic active radiation *I*, temperature *T* and the state of the enzymes, membrane pumps and pigments, *S*, determine the density of the photosynthetic rate [11].

p(t,x)=p(I(t,x),T(t,x),S(t,x)) (2)

The extinction of light in the canopy reduces photosynthesis, especially in the lower parts of the canopy and.

*Definition 5. The degree of annual photosynthetic interaction at a point is the photosynthetic production in the point x, relative to potential production in full light*.

We obtain the density of the degree of annual photosynthetic interaction, *I*_*k*_(*x*) during year k by combining Eqs 1 and 2

$$I_{k}(x)=\frac{\int_{t_{k}}^{t_{k+1}}p(I(t,\;x),\;T(t,\;x),\;S(t,\;x))\;dt}{\int_{t_{k}}^{t_{k+1}}p(I(t,\;x_{o}),\;T(t,\;x_{o}),\;S(t,\;x_{o}))\;dt},$$ (3)

where *x*_*o*_ is a point above the canopy.

Solar radiation is the source of energy in photosynthesis and the density of solar radiation flux dominates the environmental factors affecting the density of photosynthetic rate in Scots pine [11]. The absorption of light quanta by needles reduces the density of radiation flux in the canopy and the photosynthesis at the base of the canopy is strongly reduced.

*Definition 6. The shading needle mass at the point x in the stand is the needle mass per unit area above the point x in the canopy*.

We denote with *M*_*S*_ (*x*,*k*) the shading needle mass above the point *x* in the year *k*.

**Axiom 3. The shading needle mass determines the density of the annual degree of interaction**.

We formulate the above axiom as the first approximation, as follows [13]

$$I_{k}(x)=\frac{1}{1+a_{S2}M_{S}{\left(x,\;k\right)}^{0.8}}$$ (4)

where a_s2_ is a parameter.

*Definition 7. The respiration at a point in space and time is the amount of CO_2_ released in the conversion of ADP to ATP in a small volume during a short time interval divided by the product of the mass of tissue in the volume and the length of the time interval*.

We also define the density of annual amount of respiration at a point as the amount of CO_2_ released in respiration divided by the mass of the tissue in a small volume element around the point. The metabolism in leaves and fine roots is very active, while that in woody tissues is considerably less active.

**Axiom 4. The annual amounts of respiration at a point are tissue specific**.

Vegetation takes up nitrogen from the soil.

*Definition 8. Nitrogen uptake takes places when nitrogen ions penetrate the cell membrane in fine roots and enter the metabolism of the tree*.

**Axiom 5. The amount of fine roots and the concentrations of plant-available nitrogen in soil (NH_4_^+^ and NO_3_^–^) affect the nitrogen uptake by trees**.

Vegetation releases carbohydrates from roots [14,15]. The root exudates activate microbes and accelerate the decomposition of macromolecules in the soil.

*Definition 9. The root exudates are the sugar released from fine roots in the soil*.

**Axiom 6. The mass of fine roots determines the annual amount of root exudates**.

We denote with M_r_ the mass of fine roots and with E_r_ the annual amount of root exudates. The above axiom results

$$E_{r\;}=e_{r}\;M_{r}$$ (5)

where e_r_ is a parameter.

The lifetime of active cells in trees and ground vegetation is quite short, usually less than a couple of years. The proteins in the senescing tissues are an important source of nitrogen.

*Definition 10. Retranslocation takes place when vegetation decomposes proteins in senescing tissues into amino acids and transports them away*.

**Axiom 7. Retranslocation releases nitrogen from the senescing needles for use by growing tissues**.

#### Tree structure

Several hundreds or thousands trees per hectare form a pine stand. Diameters, heights and needle masses of trees vary in the stands. The variation in the tree properties can be conveniently taken into account by dividing trees into size classes.

The annual changes in the tree structure are outcomes of the growth and senescence during the growing seasons. Our ontological approach stresses the capacity of trees to control the formation of their own structure.

*Definition 11. The regulation system for the formation of tree structure determines the properties of the growing structures*.

**Axiom 8. The functioning principle of the regulation system for the formation of tree structure is to generate efficient structures at the annual level**.

The regularities determine the development of tree structure.

**Axiom 9. The regulation system for the formation of tree structure generates regularities in the structure of trees**.

*Definition 12. The functional unit of a tree is a set of structures that are connected to each other and the structures operate quite independently from the rest of the tree*.

Photosynthesis and transpiration are coupled with each other since diffusion transports carbon dioxide into leaves for photosynthesis, and water vapour out of leaves, through the stomatal pores. The loss of water in transpiration is usually hundred-fold when compared with the mass of CO_2_ captured in photosynthesis. The woody structures transport water from the soil to the needles.

**Axiom 10. The branches formed in the same year, their needles, and fine roots feeding the branch with water as well as the water transport system in branches, stem and transport roots, form functional units in Scots pine trees that we call whorls**.

**Axiom 11. The water transport capacity of branches, stem and transport roots are in balance with the transpiration from needles**.

The sapwood produced in a given year, transport water much longer time than the needles transpire. The reuse of water transporting sapwood reduces the amount of sugars and nitrogen required in the construction of water transport systems.

**Axiom 12. The water pipes leading to the dying needles are reused for the water transport to growing needles within the whorl**.

#### Annual changes in tree structure

The photosynthesis of the whorl provides raw material for growth and energy for metabolism.

**Axiom 13. The annual photosynthetic production of a whorl is used for the maintenance and root exudates of the whorl, for the growth of needles, water pipes and fine roots of the whorl, and for the growth of the top of the tree**.

We introduce mathematical notations to obtain more exact formulations of axiom 13. Let *M*_*n*_(*i*, *j*, *k*) denote the needle mass in a tree where i refers to the size class, j to the whorl and k to the year and *M*_*S*_(*i*, *j*, *k*) the shading needle mass of the whorl and *P*_*w*_(*i*, *j*, *k*) the annual photosynthetic production of the whorl.

We obtain the annual photosynthetic production of a whorl with three factors, i.e. the photosynthetic production in unshaded conditions, the needle mass of the whorl and the reduction of photosynthetic production caused by shading (Eqs 3 and 4)

$$P_{W}(i,\;j,\;k)=P(x_{o})\;M_{n}(i,\;j,\;k)\;I_{k}(x_{i\;j}),$$ (6)

where x_0_ is a point above the canopy and *x*_*i j*_ a point within the whorl *j* in the size class *i*.

We denote the branch mass with *M*_*b*_, stem mass *M*_*s*_, coarse root mass with *M*_*c*_ and fine root mass with *M*_*r*_. We obtain the annual amounts of respiration of a whorl *R*_*W*_*(i*, *j*, *k)* utilising axiom 4

$$R_{W}(i,\;j,\;k)=a_{nr}\;M_{n}(i,\;j,\;k)+a_{br}\;M_{b}(i,\;j,\;k)+a_{sr}\;M_{s}(i,\;j,\;k)+a_{cr}\;M_{c}(i,\;j,\;k)+a_{rr}\;M_{r}(i,\;j,\;k),$$ (7)

where *a*_*n r*_, *a*_*b r*,_, *a*_*s r*_, *a*_*c r*_, *and a*_*r r*_ are parameters.

We use the sapwood area of branches, stems and coarse roots as a measure of their water transport capacity. Let *G*_*n*_(*i*, *j*, *k*) denote the growth of needles, and let *G*_*Ab*_(*i*, *j*, *k*) denote the growth of sap wood area in branches, *G*_*As*_(*i*, *j*, *k*) in stem, and *G*_*A*t_(*i*, *j*, *k*) in transport roots. Since the mean life time of needles is four years in Finnish Lapland and two years in south from Finland [16], we assumed that the life time is three years in southern Finland.

Corollary, axioms 11 and 12 result in the following equations

$$G_{Ab}(i,\;j,\;k)=a_{b}(G_{n}(i,\;j,\;k)\;-\;G_{n}(i,\;j,\;k\;-\;3))$$ (8)

$$G_{As}(i,\;j,\;k)=a_{s}(G_{n}(i,\;j,\;k)\;-\;G_{n}(i,\;j,\;k\;-\;3))$$ (9)

$$G_{At}(i,\;j,\;k)=a_{t}(G_{n}(i,\;j,\;k)\;-\;G_{n}(i,\;j,\;k\;-\;3))$$ (10)

where parameters a_b_, a_s_, and a_t_ describe the sapwood requirement per unit leaf mass for branches, stem and coarse roots. If the needle mass in the whorl is decreasing, then the extra water transport capacity is lost. In other words, we assume that in this situation sapwood is slowly converted into heartwood, which cannot conduct water.

Let *M*_*T*_(*i*, *j*, *k*) denote the growth of the mass of the water transport system. It is formed by the growths in the branches, stem and transport roots. Let *L*_*b*_(*i*, *j*, *k*) denote the mean length of branches, *h*(*i*, *k*) the height of the whorl in the stem, and *b*_*t*_(*i*, *j*, *k*) the length of transport roots. Then,

$$M_{T}(i,\;j,\;k)=d_{b}\;l_{b}(i,\;j,\;k)\;G_{Ab}(i,\;j,\;k)+\;d_{s}\;h(i,\;j)\;G_{As}(i,\;j,\;k)+d_{t}\;b_{t}(i,\;j,\;k)\;G_{At}(i,\;j,\;k)$$ (11)

The parameters *d*_*b*_, *d*_*s*_ and *d*_*t*_ describe density of wood in the branches, stem and transport roots. Thus we obtain the mass of growth of the water transport system supporting each whorl from the axioms 11 and 12 utilising Eqs 6–9.

The uppermost whorls in the tree do not generate enough photosynthetic products to maintain the rapid growth of these whorls. On the other hand, the uppermost whorls should grow at a rapid pace, or the tree may lose its position to other trees in the stand. The allocation of photosynthetic products of other whorls to the three uppermost whorls is therefore beneficial to the tree.

**Axiom 14. From all the photosynthetic products, the regulation system for the formation of tree structure allocates a tree-size dependent share for the development of the top of the tree**.

Let *T*_*A*_*(i*, *j*, *k)* denote the amount of carbohydrates allocated to the top of the tree. We approximate the allocation to the top with the following function:

$$T_{A}(i,\;j,\;k)=\frac{a_{t1}(P_{W}(i,\;j,\;k)\;-\;R(i,\;j,\;k))}{1+h(i,\;j)/a_{t2}},$$ (12)

where *a*_*t 1*_ and *a*_*t 2*_ are parameters. Axiom 13 results in a carbon balance equation that is one of the cornerstones of our simulations.

The annual photosynthetic production of a whorl *P*_*w*_ is used to respiration *R*_*w*_, to root exudates *E*, allocation to the top *T*_*w*_ to growth of needles, *G*_*n*_, to water transport system *M*_*T*_ and to growth of fine roots *M*_*r*_. The carbon balance equation is

$$P_{W}(i,\;j,\;k)\;-\;R_{W}(i,\;j,\;k)\;-\;E_{r}(i,\;j,\;k)\;-T_{A}(i,\;j,\;k)=a_{ngr}\;G_{n}(i,\;j,\;k)+a_{wgr}M_{T}(i,\;j,\;k)+a_{rgr}\;M_{r}(i,\;j,\;k)$$ (13)

The parameters *a*_*n gr*,_ *g*_*r*_, *a*_*w gr*_ and *a*_*r gr*_ are chemical conversion coefficients from sugars to needle, wood and fine root tissues.

The metabolism within tree structures is driven by nitrogen rich substances, thus a sufficient amount of nitrogen is needed for growth. The growth requires that the nitrogen requirement of all new tissues is met.

**Axiom 15. The product of the concentration of plant-available nitrogen and the amount of fine roots in the soil determine the nitrogen uptake by tree roots**.

Let *U* denote the nitrogen uptake, *M*_*r*_ (*i*, *j*, *k*) the root mass and *N*_*a*_*(k)* the concentration of plant-available nitrogen in the soil in the year k. Axiom 15 results in

$$U(i,\;j,\;k)=u\;M_{r}(i,\;j,\;k)\;N_{a}(k),$$ (14)

where *u* is a parameter.

The metabolism of vegetation is based on the action of enzymes, membrane pumps and pigment complexes that are proteins.

**Axiom 16. The annual uptake and retranslocation of nitrogen are utilised for the synthesis of proteins in the new tissues during the year**.

**Axiom 16 results in the nitrogen balan**ce equation

$$n_{n}\;G_{n}(i,\;j,\;k)+n_{w}\;M_{T}(i,\;j,\;k)+n_{r}\;M_{r}(i,\;j,\;k)=\;U(i,\;j,\;k)+(\;n_{n}\;-n_{L})\;G_{n}(i,\;j,\;k\;-\;3),$$ (15)

where *n*_*n*_, *n*_*w*_, n_r_ are nitrogen concentration parameters and the parameter c is for retranslocation of nitrogen.

The regulation system of formation of tree structure is active during the growth of needles, woody components and of fine roots.

**Axiom 17. The action principle of the regulation system for the formation of tree structure is to fulfil simultaneously the carbon and nitrogen balance equations**.

The above action principle is a direct consequence of our ontological approach and the cornerstone of our simulations.

The carbon and nitrogen balance equations include two unknowns: the growths of needles and fine roots. We can solve these from the two equations and we obtain the growths for each whorl in the stand. The balance between photosynthesis, nitrogen uptake and water transport within trees plays a very important role in the development of tree structure.

The regulation system for the formation of tree structure accelerates the height growth of trees growing in the shade cast by bigger trees.

**Axiom 18. The ratio between the height and diameter growth is constant for open grown trees and the interaction with other trees accelerate height growth**.

For describing the acceleration of height growth caused by the shading of other trees in the stand, we introduce the mean annual degree of interaction *I*_*T*_*(i*, *k)* experienced by the tree size class i during the year k

$$I_{T}(i,\;k)=\frac{\sum_{j=1}^{k}I_{p}(x_{i\;j})\;M_{n}(i,\;j,\;k)}{\sum_{j=1}^{k}\;M_{n}(i,\;j,\;k)},$$ (16)

where *I*_*k*_ is defined in Eq (3).

Let Δ*h* denote the height growth and Δ*r* the diameter growth. We introduce the relationship between the height and radial growth, and the mean degree of interaction as follows

$$\Delta h(i,\;k)=a_{h1}\;\Delta r(i,\;k)\;(1+a_{h2}\;{\left(1+I_{T}(i,\;k)\right)}^{4}\;),$$ (17)

where *a*_*h1*_ and *a*_*h2*_ are parameters.

The branches in a whorl expand their length annually. This expansion is under the control of the regulation system for the formation of tree structure.

**Axiom 19. The action principle of the regulation system for the formation of tree structure on the length growth of branches is to maintain a constant needle density in the crown**.

Corollary, the length growth of branch is the solution of the following equation

$$\frac{G_{n}(i,\;j,\;k)}{2\;\pi \;\Delta L_{B}(i,\;j,\;k)\;L_{B}(i,\;j,\;k)\;\Delta h(i,\;k)}=\rho_{n},$$ (18)

where Δ*L*_*B*_ is the length growth of a branch, *L*_*B*_ is the length of the branches in the whorl and *ρ*_*n*_ is needle density.

We introduce a small and technical assumption: a whorl dies when its length growth is less than 1 cm and a tree dies when all whorls in the tree have died.

In addition to transporting water to the above growing needles, the stem has also to bear the mechanical load of aboveground biomass. The mechanical stresses are the highest in the lower parts of trunk. To balance these loads the regulation system of tree structure grows an enlargement at the stem base.

*Definition 13. Stem base swelling is the enlargement of stem near ground*.

**Axiom 20. The stem base swelling is largest at the ground level and it disappears rapidly when moving upwards along the stem**.

Let Δ*A*_*E*_ (*i*, *j*, *k*, *h*_*a*_) denote the growth of the area of the stem base swelling at height h_a_ grown during year *k*, utilizing sugars from whorl *j* in the tree in the size class *i*. We use a rough approximation to introduce the stem base expansion

$$\begin{array}{l} \Delta A_{E}(i,\;j,\;k,\;h_{a})=\{\begin{array}{l} 0,\;if\;h_{a}\;\geq \;h(i,\;k)/10\;\\ a_{b1}\;(1\;-\;\frac{h/10}{h(i,\;k)})\;G_{As}(i,\;j,\;k)\;\;if\;h_{a}\;\leq \;h(i,\;k)/10 \end{array} \end{array}$$ (19)

where *a*_*b 1*_ is a parameter.

### Ground vegetation

The Scots pine forest floor is covered by ground vegetation, which usually consists of dwarf shrubs, herbs and mosses. The living biomass of the ground vegetation is often rather small, but when the trees are not reducing light too strongly at the ground level, the annual growth of new leaves per unit area is of the same magnitude as that of trees. As a rough approximation, the chemical composition of ground vegetation can be assumed to be close to that of the trees.

**Axiom 21. The shading by the tree canopy reduces the mean density of annual photosynthetic production of ground vegetation**.

**Axiom 22. The shading within ground vegetation reduces the mean density of annual photosynthetic production of ground vegetation**.

We approximate the photosynthesis of ground vegetation, *P*_*G*_ as follows

$$P_{G}(M_{nG})=p_{g}\;f_{S}(M_{sn})\;M_{nG}\;(1\;-\;\frac{M_{nG}}{M_{nG}\;-\;g_{i}}),$$ (20)

where *M*_*n G*_, is the leaf mass of ground vegetation, *M*_*rG*_ the fine root mass of ground vegetation, and *M*_*s n*_ the needle mass of the tree stand and function *I*_*k*_ is defined in Eq (4) and *p*_*g*_ and *g*_*i*_ are parameters.

The ground vegetation controls the formation of its structure.

**Axiom 23. The regulation system for the formation of structure determines the structural properties of the ground vegetation**.

The carbon and nitrogen balance equations that resulted from our ontological approach played an important role in the analysis of the tree structure formation. We take into consideration that the water transport system is so small in ground vegetation that we can omit it. In this way, we obtain the carbon and nitrogen balance equations

$$P_{G}-R_{G}=a_{ngr}\;M_{nG}+a_{rgr}\;M_{rG}$$ (21)

$$(1\;-\;c)\;n_{n}\;M_{nG}+n_{r}\;M_{rG}=u\;M_{rG}\;N_{a}.$$ (22)

where a_n gr_ and a_r gr_ are conversion coefficients from sugars to leaves and roots.

**Axiom 24. The functioning principle of the regulation system for the formation of ground vegetation structure is that the new structure fulfils the carbon and nitrogen balance equations**.

We obtain the annual leaf and fine root growths of ground vegetation as the solution of the carbon and nitrogen balance equations.

### Forest soil

#### Metabolism

The large biopolymers in the plant litter, such as cellulose and proteins, are not directly available for microbes or trees. Microbes decompose the organic macromolecules by extra-cellular enzymes [17–19] to small molecules such as sugars, amino acids, fatty acids and various other aliphatic and aromatic compounds [20–24]. Microbes take up these small molecules. In addition, small amounts of humic substances are formed. The decomposition of organic matter in soil is a slow process, requiring decades. The lifetime of humus is very long and varies according to its chemical properties [25]. The carbon fluxes connected with humic substances are, however, so small that we neglect them from the carbon dynamics. We treat soil per unit area (m^–2^) according the traditions of soil science.

Most of the nitrogen available for trees is loosely adsorbed on the surface of soil particles as ammonium ions. When microbes use amino acids to produce ATP, they emit ammonium ions. The lifetime of ammonium in the soil is short, only about 30 days [26].

Litter fall is the main carbon and nitrogen input into forest soil, while nutrient uptake causes the main nitrogen flow out of the soil. In addition, deposition and fixation from the atmosphere, leaching of nitrogen to ground water and emission of nitrogenous gases to the atmosphere connect the forest soil with its environment. Fig 1 shows carbon compound fluxes between the main pools in the forest ecosystem. Circulation of nitrogen within the soil is an additional complication in the soil nitrogen fluxes compared to carbon fluxes (Fig 2).

*Definition 14. Macromolecules are biopolymers, formed by several similar carbon rich units*.

**Axiom 25. Five types of macromolecules (cellulose, lignin, lipids, starch and proteins) dominate the soil**.

In addition, there is a very passive component of humus compounds that decay in the time scale of millennia [25].

Large carbon containing molecules are polymers of small carbon containing monomer compounds and there are characteristic chemical bonds between the basic carbon units. Special enzymes are able to cleave each bond type.

*Definition 15. The microbe specialised to the decomposition of the macromolecule is the microbe specialised to secrete enzyme that catalyse the cleavage of the some five macromolecules*.

#### Analysis of the steps in soil

In contrast to vegetation, the soil microbes have no clear annual cycle in their metabolism. This is why we use continuous time in the formulation of the soil related equations. Long reaction pathways, in which the molecular structure of compounds changes in metabolic reactions, characterizes carbon (Fig 1) and nitrogen (Fig 2) flows in the soil. The metabolism of each step in the chains is quite well understood, therefore we can base the axioms and equations on well established physiological knowledge. We formulate the knowledge as axioms following the Newtonian example. We also, implicitly, assume that the models that describe the relationships between process rates and environment, do not introduce any major inaccuracies into the simulations.

Thermal movement of macromolecules in the soil solution generates contacts between macromolecules and the enzymes in the soil solution resulting in cleaving of the molecule.

*Definition 16. The cleaving per square meter of each macromolecule type is the ratio between the mass of cleaved macromolecules per square metre during a short time interval and the length of the time interval*.

The cleaving rate of macromolecules changes during the development of the ecosystem.

**Axiom 26. The product of the concentrations of the given macromolecule type and its bond specific cleaving enzyme determine the cleaving macromolecule rate per square metre**.

Let, *C*_*L n*_ be the density (g m^–2^) of large carbon molecules of the type *n*, *E*_*n*_ the density (g m^–2^) of the enzyme cleaving molecules of type *n* and *s*_*p n*_ the cleaving rate of macro molecules of type n Then according the Axiom 26 we obtain

$$s_{pn}=b_{1n}\;E_{n}\;C_{Ln},$$ (23)

where *b*_*l n*_ are parameters.

The cleaving of macromolecules results in small carbon containing compounds that microbes transport through the cell membrane with membrane pumps.

*Definition 17. The uptake rate per square metre of small molecules by microbes is the mass of small molecules per square meter penetrating the microbial cell membrane during a short time interval divided by the length of the interval*.

The uptake rate is dynamic and it varies during the ecosystem development.

**Axiom 27. The uptake rate of small molecules per square metre depends on their concentration in the soil solution**.

Let *C*_*Sn*_ be the small molecule concentration in the soil solution. Axiom 27 states that

$$u_{pn}=b_{2n}\;C_{Sn},$$ (24)

where *b*_*2n*_ is a parameter.

The microbe population in the soil is dynamic, the population grows and dies.

*Definition 18. The growth rate of microbes per square meter is the mass of new microbes formed per square meter during a short interval divided by the length of the interval*.

The growth rate per square meter describes in exact formulation the formation of new microbes.

**Axiom 28. The product of the mass of the microbes of type n and the amount of small molecules of type n in microbes determines the growth rate per square metre of microbes of type n**.

Let, *g*_*mn*._, be the growth rate of microbes of type n per square meter and *M*_*Mn*_ the mass of microbes of type *n*. The Axiom 28 determines the growth rate of microbes per square meter, *g*_*mn*._

$$g_{mn}=b_{4n}\;C_{Sn}\;M_{Mn},$$ (25)

where *b*_*4n*_ is a parameter.

The lifetime of microbes is limited.

*Definition 19. The death of a microbe takes place when the microbial cell membrane loses its capacity to control the flow of material through its membrane*.

*Definition 20. The death of microbes per square meter is the mass of dying microbes per square meter during a short time interval divided by the length of the interval*.

**Axiom 29. Microbes have specific tendencies for dying**.

Exact formulation results in the concept of death rate per square metre. Let *d*_*mn*_ the death rate of microbes per square metre and *M*_*Mn*_ be the mass of microbes of the type *n*. The death rate of microbes according to the Axiom 29 is

$$d_{mn}=b_{5n}\;M_{Mn},$$ (26)

where *b*_*5n*_ (n = 1, 2, 3, 4, 5) are parameters.

Microbes utilize the amino acids in the soil solution in their metabolism.

*Definition 21. The uptake rate per square meter of amino acids by microbes is the mass of amino acids penetrating the microbial cell membrane per square metre during a short time interval divided by the length of the interval*.

In mathematical terms we obtain the concept amino acid uptake rate.

**Axiom 30. The different microbe types have equally effective intake rates of amino acids and the availability of amino acids determines the uptake per square meter**.

Let *u*_*pn*_, be the amino acid uptake rate per square meter by type n microbes and *C*_*SM 1*_ the amino acid content int he soil. According the Axiom 30 we obtain

$$u_{pn}=c_{1}\;\frac{M_{Mn}}{\sum_{j=1}^{5}M_{Mj}}C_{SM1},$$ (27)

where *c*_*1*_ is a parameter.

Microbes utilize the up-taken amino acids either to enzyme synthesis or to energy needs.

*Definition 22. The enzyme emission per square meter by microbes is the amount of emitted enzyme per square meter during a short time interval divided by the length of the interval*.

**Axiom 31. The shares of each microbe types and the amino acids in microbes determine the emission per square meter of the enzyme type splitting macromolecule**.

Let *q*_*en*_, be the emission rate per square meter of enzyme splitting of type n macro molecules, *C*_*SM1*_ the amino acid content in the soil microbes and M be the mass of microbes of the type *n*.

According the axiom 31 we obtain

$$q_{en}=c_{2}\frac{M_{Mn}}{\sum_{j=1}^{5}M_{Mj}}C_{SM1},$$ (28)

where *c*_*2*_ is a parameter.

The extracellular enzymes are proteins and as such they are vulnerable to decomposition by other extracellular enzymes.

*Definition 23. The decomposition per square meter of enzymes is the amount of decomposed enzymes over a short time interval divided by the length of the short period*.

The availability of material and concentration of protein splitting enzymes determine the decomposition of enzymes.

**Axiom 32. The product of the concentration of enzyme type n and the concentration of enzymes decomposing it determine the decomposition per square meter of the enzyme**.

We denote the decomposition rate per square meter of enzyme, type n, with *s*_*n 1*_ and with E_n_ the concentration of extracellular enzyme splitting the macromolecules, type n. In addition, we number the macromolecules starting from proteins. Axiom 32 results

$$s_{en}=c_{5}\;E_{1}\;E_{n},$$ (29)

where *c*_*5*_ is parameter.

The microbes need energy for their metabolism.

*Definition 24. The microbial respiration rate per square meter is the mass of CO_2_ emitted by microbes per square metre over a short time interval divided by the length of the interval*.

We use the term microbial respiration rate per square metre in the exact mathematical formulations.

**Axiom 33. The small carbon compound concentration in microbes determines the microbial respiration rate per square metre. Accordingly, the microbial respiration rate r_m_ is**

$$r_{m}=c_{4}\;\sum_{j=1}^{5}e_{j}\;C_{SMj}$$ (30)

where *c*_*4*_ and *e*_*j*_ are parameters, the latter converting carbon compounds to CO_2_.

The microbes also use amino acids for their energy needs. Then microbes emit the nitrogen in proteins used for energy release as ammonium ions.

*Definition 25. The ammonium release per square meter from microbes is the amount of released ammonium ions per square metre during a short time interval divided by the length of the interval*.

Let *n*_*r*_, be the ammonium release rate square meter it depends on the availability of raw material.

**Axiom 34. The amino acid content in the microbes determines the ammonium release rate per square metre**.

Accordingly,

$$n_{r}=c_{3}\;C_{SM1},$$ (31)

where *c*_*3*_ is a parameter.

### Accumulation of changes in a forest ecosystem

The definitions and axioms deal with changes either in tree and ground vegetation structure or in the amounts of chemical compounds or microbes in the soil. These changes accumulate during the ecosystem development and we obtain the development of trees and ground vegetation structures and of properties of soil during prolonged periods by adding the changes described above. We base the combination of the axioms in the mathematical analysis on the principle of conservation of mass.

#### Accumulation of annual changes in trees

Trees operate in the annual time scale, for example, they form annual whorls and tree rings. The changes in needle mass are the driving forces of tree development. We obtain the needle mass of the whorl by adding the new needles and removing the senescent ones (we assume that needles live for three years)

$$M_{n}(i,\;j,\;k+1)=M_{n}(i,\;j,\;k)+G_{n}(i,\;j,\;k)\;-\;G_{n}(i,\;j,\;k-3)$$ (32)

We also obtain the sapwood area by adding the new wood to the old one. Let *A*(*i*, *j*, *k*), denote the cross-sectional saparea in the i^th^ size class at the j^th^ whorl during the year k. The cross-sectional sapwood area of the stem at the top of the tree is zero, thus *A*(*i*, *j*, *k*) = 0. The area, *A*(*i*, *j*, *k*) is obtained recursively by adding new water pipes for the above whorls in the area during the previous year

$$A(i,\;j,\;k)=A(i,\;j,\;k-1)+\sum_{jj=j+1}^{k}G_{As}(i,\;jj,\;k).$$ (33)

We assume that the stems have a circular cross-section, and obtain the diameter of the stems with the well-known relationship between the area of a circle and its diameter.

We calculate the heights of the trees in the year *k*+1, *h*(*i*, *k*+1), from the height during the year *k* and from the height growth

h(i,k)=h(i,k−1)+Δh(i,k). (34)

We treat the branches in a similar way

$$L_{B}(i,\;j,\;k)=L_{B}(i,\;j,\;k-1)+\Delta L_{B}(i,\;j,k).$$ (35)

#### Ground vegetation

Annual plant structures have a dominating role in the ground vegetation and there is no major accumulation of material into the ground vegetation. Thus the annual solutions of the carbon and nitrogen balance equations provide the leaf and fine root masses in the ground vegetation.

#### Accumulation of changes in soil

The annual time step is characteristic for the development of tree structure. In contrast, the microbes in the soil react more to the prevailing environmental factors. Thus, continuous time is more appropriate in the analysis of soil development and we formulate the changes in the pools of organic matter in the soil as differential equations based on conservation of mass and nitrogen.

We treat separately the five macro molecule types (proteins, sugars, starch, lipids and lignum) and we indicate the macro molecule type with subscript n, when needed. The litter fall *L*_*n*_ and death of microbes *d*_*M n*_ increase the amount of macromolecules *C*_*Ln*_ in the soil and cleaving *s*_*pn*_ decrease it

$$\frac{dC_{Ln}}{dt}=L_{n}+d_{Mn}-s_{pn}.$$ (36)

The cleaving of macromolecules *s*_*P*_ increase and microbial uptake *u*_*p*_ decrease the small molecules in soil water *C*_*S*_

$$\frac{dC_{S\;}}{dt}=s_{p}\;-\;u_{p}$$ (37)

Microbial uptake *u*_*p*_ increases the small molecules in microbes. Microbes utilize the small carbon molecules in their metabolism for the production of ATP resulting respiration *r*_*m*_ and for growth of new microbes *g*_*m*_ (Fig 1).

$$\frac{dC_{M}}{dt}=u_{p}\;-\;g_{m}\;-\;r_{m}$$ (38)

Growth *g*_*M*_ increase, and death *d*_*M*_ and emission of enzymes *q*_*e*_ decrease large molecules *M*_*M*_ in microbes

$$\frac{dM_{M}}{dt}=g_{M}-d_{M}-q_{e}$$ (39)

Nitrogen flows in forest ecosystem form two loops; one in the soil and the other combining the vegetation and soil. The outer loop operates on annual time scale. Here we treat the smaller loop dealing with the nitrogen flows in the soil.

Proteins in the litter fall *L*_*1*_ and death of microbes *d*_*m*_ feed the protein pool in the soil *C*_*L 1*_ and decomposition of proteins *s*_*p1*_ decrease it

$$\frac{dC_{L1}}{dt}=L_{1}+d_{m1}\;-\;s_{p1}$$ (40)

Decomposition of soil proteins *s*_*p 1*_ and of enzymes *d*_*e 1*_ increase the amino acid pool *C*_*s 1*_ in the soil and uptake *u*_*p1*_ by microbes decrease it

$$\frac{dC_{s1}}{dt}=s_{p1}+d_{e1}-u_{p1}$$ (41)

Microbial uptake of amino acids *u*_*p1*_ increase and growth *g*_*m1*_ and release of ammonium *n*_*r*_ decrease the amino acid pool C_SM 1_ in the microbes

$$\frac{dC_{SM1}}{dt}=u_{p1}\;-\;n_{r}\;-\;g_{m1}$$ (42)

Growth of *microbes g*_*m 1*_ ncrease, emission of enzymes *q*_*e*_ and death of microbes *d*_*e*_ decrease proteins in microbes *M*_*M*_

$$\frac{dM_{M}}{dt}=g_{m1}\;-\;q_{e}\;-\;d_{e}$$ (43)

The microbial emission of enzymes *q*_*e n*_ and decomposition of enzymes *d*_*e n*_ change the extra cellular enzymes *E*_*n*_ in the soil

$$\frac{dEn}{dt}=q_{en}-d_{en}$$ (44)

The equation is carbon-molecule specific.

#### Connections between vegetation and soil

*Definition 26. The connections between vegetation and soil are the effects of vegetation on the properties of soil and the effects of soil on vegetation*.

**Axiom 34. Nitrogen and carbon compound fluxes convey the connections between vegetation and soil in forest ecosystem**.

The amount of macromolecules in the forest soil is increased by senescent trees and ground vegetation organelles and dead microbes, and decreased by the cleaving action of extracellular enzymes. We denote with *L*_n_ the litter fall of large carbon molecules of type n, per square meter. Needles are the main component in the litter, but litter also includes the wood from dead branches and trees. We consider the changes in the forest soil organic matter in Eq 35.

We have to combine two time scales, i.e. annual and continuous time. We combined the annual fluxes changing the amount of nitrogen ions in the soil, i.e. nitrogen uptake by vegetation (Eqs 15 and 22), release by microbes *n*_*r*_, deposition *d*_*ep*_, nitrogen fixation *n*_*f*_, leaching l_*e*_ and volatilization *e*_*v*_ by formulating the equations in the annual scale. Then the conservation of nitrogen results

$$\frac{dN_{a}}{dt}=-\;u\;\sum_{i=1}^{5}\sum_{j=1}^{k}M_{r}(i,\;j,\;k)\;N_{a}(k)\;-\;u\;M_{rG}\;N_{a}(k)+n_{f}+d_{ep}\;-\;l_{e}\;-\;e_{v}+n_{r}.$$ (45)

The nitrogen ion content in the soil plays an important role in the dynamics of the forest ecosystem because it is crucial for the allocation of sugars to roots.

## Mathematical analysis of the behaviour of forest ecosystem

### Simulation approach

We defined the concepts dealing with the most important features of vegetation and soil and we also introduced the axioms characterising the changes in trees and ground vegetation at annual level and the behaviour of soil at instantaneous responses. In the analysis, our primary interest is to investigate time scales corresponding to the rotation period in boreal forest management. Thus we should accumulate the annual or instantaneous changes that our concepts and axioms are dealing with, during the rotation time. This is analogous with Newton's transition from the action of gravity at point level to the circulation of planets around the sun.

We are unable to solve the equations in closed-form, as Newton did. Instead we have to use numeric simulation methods. We then predict the behaviour of the forest ecosystem from a given initial state over several decades, up to the rotation period. The regulation system for the formation of tree structure in Scots pine operates at an annual time scale. This is why we repeatedly add the yearly changes in masses of trees to the values at the beginning of the given year. In addition, we determine the annual values of sapwood area, tree height and branch length. We calculate the changes in soil with a short time step (1/100 years).

The numeric simulation requires values for all masses, areas, lengths and heights in the equations in the beginning of the simulations.

*Definition 27. We call the set of all masses, areas, lengths and heights in the beginning of the simulation as the initial state*.

*Definition 28. The constants in the equations are parameters*.

#### Measurements

We planned and constructed the SMEAR II measuring station [27] to measure all relevant materials and energy fluxes and material pools in the forest ecosystem around the station. SMEAR II is surrounded by even-aged Scots pines growing on a shallow coarse-textured soil of middle fertility overlying bedrock. Our concepts and axioms concern carbon and nitrogen fluxes in forest ecosystems. We measure these fluxes at SMEAR II and therefore, the theoretical considerations and measurements are coherent with each other.

The measurement of each tree in the stand is impractical and thus, we obtained the development of the trees from samples. We sampled the stand around SMEAR II in 2001, in such a way that the large trees were more probable to be included in the sample. We formed five size classes according to the diameter in the beginning in such way that the 5% of biggest trees form the biggest size class, next 15% the second, next 30% third one, next 30% and the remaining 20% the fifth one. We measured retrospectively the annual diameters and heights until the age of five years for 25 trees (five trees per size class). We then determined the measured diameters and heights as the annual mean diameters and heights in each size class.

#### Initial state

As the initial state of the stand, we used the initial mean diameters and heights at the age of five years from the retrospective measurements. We obtained the needle masses at the initial state from empirical regressions between sapwood area and needle mass in small seedlings. We also applied empirical regressions to obtain the initial branch lengths.

We lack measurements of the soil properties when the stand was regenerated by sowing after prescribed burning in 1961. We assumed that the soil properties change slowly. We measured the pools of proteins, cellulose, lignin, starch and lipids in 2007 in the stand around SMEAR II and we used the obtained values as the soil initial state.

#### Parameter values

The axioms and their formulation with equations introduce quite a few parameters and we need their values for the simulations. We were unable to obtain the values of the parameters from theoretical thinking as sometimes in physics. We used several sources of information to obtain the values of the parameters (Table 1). The measurements at SMEAR II were very useful for determining the values dealing with gas exchange, structure of trees and soil properties. We used literature values for the chemical properties of macro molecules. We applied static behaviour criteria to obtain the values of the parameters involved in the decomposition of organic material in the soil. There were 10 parameters that we were unable to determine with the above criteria. They address the stem base expansion, extinction of light in the canopy, height growth, the allocation of sugars to the top of the tree, shading within ground vegetation and the decomposition of proteins in the soil. We determined these parameter values from the behaviour of the predictions characterised by the concepts and axioms. NewtonForest predicted the behaviour of all state variables over the years. However, there was one parameter specific state variable that was very sensitive to the parameters to be estimated and in addition, there was one value of the parameter that resulted static behaviour of the state variable in question. This enabled the estimation of the parameters with the method of trial and error. We collected all parameters values into Table 1.

**Table 1:** List of parameters their values, units, equations in the text and source of information

Parameter	Value	Unit	Equation	Source
P	13.6	g(CO_2_) g(dry weight) –1	1,6	SMEAR II
a_s 1_	400	cm g(dry weight) –1	2	[43]
a_s 2_	0.00000027	g(dry weight)^–1^	4	**Simulations with MicroForest**
e_r_	15	g(CO_2_) g(dry weight) –1	5	**Simulations with NewtonForest**
a_n r_	2	g(CO_2_) g(dry weight) –1	6	SMEAR II
a_b r_	0.02	g(CO_2_) g(dry weight) –1	6	SMEAR II
a_s r_	0.02	g(CO_2_) g(dry weight) –1	6	SMEAR II
a_t r_	0.02	g(CO_2_) g(dry weight) –1	6	SMEAR II
a_r r_	16	g(CO_2_) g(dry weight) –1	6	**Simulations with NewtonForest**
a_b_	0.03	cm^2^ g(dry weight) –1	7	SMEAR II
a_s_	0.033	cm^2^ g(dry weight) –1	8	SMEAR II
a_t_	0.015	cm^2^ g(dry weight) –1	9	SMEAR II
a_b 1_	1.5		10	**Simulations with NewtonForest**
d_b_	0.4	g(dry weight) cm –3	11	SMEAR II
d_s_	0.4	g(dry weight) cm –3	11	SMEAR II
d_t_	0.2	g(dry weight) cm –3	11	SMEAR II
a_t 1_	0.85		12	**Simulations with NewtonForest**
a_t 2_	500	cm	12	**Simulations with NewtonForest**
a_n gr_	1.35	g(sugar) g(dry weight) –1	13, 26	[44]
a_w gr_	1.3	g(sugar) g(dry weight) –1	13	[44]
a_r gr_	1.35	g(sugar) g(dry weight) –1	13,	[44]
n_n_	0.0134	g(N) g(dry weight) –1	15	SMEAR II
n_w_	0.00024	g(N) g(dry weight) –1	15	SMEAR II
n_r_	0.0134	g(N) g(dry weight) –1	15	SMEAR II
c	0.575	g(N) g(dry weight) –1	15	SMEAR II
u	0.002	m^2^ g(dry weight) –1	22	**Simulations with NewtonForest**
a_h 1_	50		17	[43]
a_h 2_	2400		17	**Simulations with NewtonForest**
*ρ*	0.00025	g(dry weight) cm –3	18	SMEAR II
p_g_	30	g(sugar) g(dry weight) –1	20	SMEAR II
g_1_	80	g(dry weight) m^–2^	20	SMEAR II
b_1 1_	0.00043	m^2^ a^–1^ g^–1^	23	Stable behaviour
b_1 2_	0.0035	m^2^ a^–1^ g^–1^	2	Stable behaviour
b_1 3_	0.0006	m^2^ a^–1^ g^–1^	23	Stable behaviour
b_1 4_	0.0012	m^2^ a^–1^ g^–1^	23	Stable behaviour
b_1 5_	0.0002	m^2^ a^–1^ g^–1^	23	Stable behaviour
b_2 1_	1	a^–1^	24	Stable behaviour
b_2 2_	1	a^–1^	24	Stable behaviour
b_2 3_	1	a^–1^	24	Stable behaviour
b_2 4_	1	a^–1^	24	Stable behaviour
b_2 5_	1	a^–1^	24	Stable behaviour
b_4 1_	0.004	a^–1^	25	Stable behaviour
b_4 2_	0.0003	a^–1^	25	Stable behaviour
b_4 3_	0.0003	a^–1^	25	Stable behaviour
b_4 4_	0.00006	a^–1^	25	Stable behaviour
b_4 5_	0.0003	a^–1^	25	Stable behaviour
b_5 1_	0.004	a^–1^	26	Stable behaviour
b_5 2_	0.007	a^–1^	26	Stable behaviour
b_5 3_	0.003	a^–1^	26	Stable behaviour
b_5 4_	0.004	a^–1^	26	Stable behaviour
b_5 5_	0.002	a^–1^	26	Stable behaviour
c_1_	1	a^–1^	32	Stable behaviour
c_2_	0.55	a^–1^	33	Stable behaviour
c_3_	6.7	a^–1^	34	Stable behaviour
c_4_	10	a^–1^	35	Stable behaviour
c_5_	0.00043	a^–1^	36	Stable behaviour

We have no measurements available on soil dynamics and therefore, the determination of the parameters dealing with soil properties is problematic. We found in simulations that there were soil state variables that obtained reasonable values within a rather narrow. In addition, there was parameters that resulted into very stable state variable values. We used the method of trial and error to find the parameter values that resulted in stable behaviour of the state variables. This stable behaviour is a strange characteristic feature of NewtonForest generated by the fact that only nitrogen compounds are important for the dynamics of the soil.

### Sensitivity analysis

NewtonForest simulates the value of over 10 000 state variables covering the needles of each whorl, the dimensions of trees and the carbon macromolecule pools in the soil. In a sensitivity analysis, we focused on the most important aspects of the simulations and thus, we selected the diameter of the biggest trees, stand needle mass and stand volume together with the protein pool in the soil for our analysis.

We estimated the values for 10 parameters from the model behaviour. The estimated parameters are: shading parameter a_s2_ (Eq 4), level of allocation of photosynthates to the top of the tree *a*_*t 1*_ (Eq 12), reduction of the allocation to tree top caused by tree height *a*_*t 2*_ (Eq 12), coefficient for root exudates e_r_ (Eq 5) The ratio between tree height and diameter for open grown trees *a*_*h 1*_ (Eq 17). interaction parameter *a*_*h 2*_ (Eq 17), stem base expansion parameter *a*_*b 1*_ (Eq 19), level of photosynthesis by ground vegetation *p*_*g*_ (Eq 20), saturation of photosynthesis in ground vegetation *g*_*i*_ (Eq 20), and cleaving of proteins in soil *b*_*1 1*_ (Eq 23). In the sensitivity analysis, we increased and decreased the parameter values by 5% one by one.

The simulations with NewtonForest require the initial state, i.e. the values of all state variables at the beginning of the simulation. Thus, we selected the stand around the SMEAR II for the sensitivity analysis and simulated the development of the ecosystem during 50 years. We determined the relative sensitivity by dividing the change in the state variable at the end of the simulation with the original value of the state variable. We collected the relative sensitivities of the four chosen state variables in the Table 2.

**Table 2:** The relative change (%) in the state variables (stand needle mass, diameter of biggest size class, stand volume ground vegetation leaf mass and proteins in the soil) over 50 years as a response to 5% increase in the parameter values.

Parameter (Equation)	State variables				
	Needle (%)	Diameter (%)	Volume (%)	Ground v. (%)	Proteins (%)
*a*_*S 2*_ (9)	4.47	2.29	8.1	–1.5	–1.9
*a*_*t 1*_ (10)	0.20	–1.3	2.5	–1.1	–0.11
*a*_*t 2*_ (10)	0.04	–0.5	1.1	0.01	–0.04
*e*_*r*_ (5)	4.0	0.75	4.9	–3.7	–0.97
*a*_*h 1*_ (17)	–0.56	1.9	11.9	29.8	–0.34
*a*_*h 2*_ (17)	–0.94	–0.87	1.2	4.7	–0.06
*a*_*b 1*_ (8)	0.16	1.46	0.36	–0.53	–0.05
*p*_*g*_ (20)	–11.8	–6.1	–22.4	99.1	1.2
*g*_*i*_ (20)	–3.6	–2.8	–11.7	16.0	0.35
*b*_*1 1*_ (23)	11.3	5.4	22.7	20.3	–2.1

The simulations showed some differences in the responses. For example, the changes in the parameters affecting stem base enlargement had hardly any effect on the behaviour of the simulations whereas the modifications in the parameters concerning the ratio between tree height and diameter, ground vegetation photosynthesis and in the cleaving of proteins in soil caused the most notable changes in the state variables (Table 2). Nevertheless, the sensitivity analysis suggests that very regular behaviour is characteristic for the development of diameters and heights. This regular behaviour can be reached only within a very narrow range of the protein decomposition parameter. Surprisingly, the shading within ground vegetation played also an important role in the beginning of the ecosystem development.

### Carbon and nitrogen fluxes at SMEAR II

**Fig 3:**
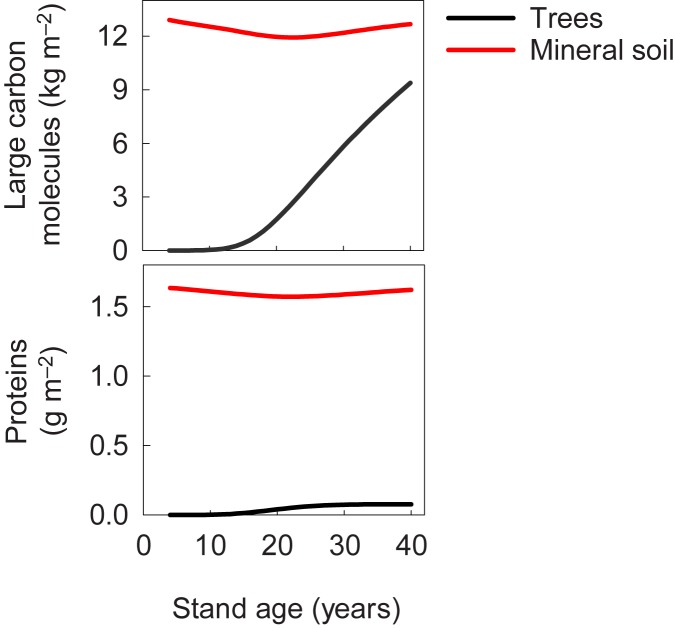
Simulated development of carbon compounds (upper panel) and proteins (lower panel) in trees and soil in the ecosystem around the SMEAR II measuring station.

We simulated the development of the ecosystem around SMEAR II. The simulations were in agreement with the versatile measurements done to characterise the carbon and nitrogen fluxes and pools during stand development from small seedlings to young trees. Fig 3 demonstrates the behaviour of simulated carbon and nitrogen pools starting from the establishment of the stand.

The simulations of the stand around SMEAR II provide important understanding of the dynamics of forest ecosystems, but they are not a real test of the concepts and axioms, since we forced the simulations to fit the measurements when we determined some of the parameter values. For a real test of our Newtonian theory of forest ecology, we need additional data that is not used to force the simulations to resemble the measured values.

The code implementing the model in R as well as the datasets are publicly available at http://urn.fi/urn:nbn:fi:csc-kata20170426111026291469.

## Testing the theory

### Test sites

There is probably no other boreal ecosystem that is measured as intensively as the stand around SMEAR II. In order to test our approach in other stands, we have to do additional measurements. We need measurements that cover the development of the system after the stand-replacing disturbance when the stand was born. Scots pine trees form clear annual whorls and tree rings that enable the retrospective measurements of the diameters and tree heights during the stand development with relative ease. The measurements are problematic only in the very early years of stand development. The retrospective measurements of the carbon and nitrogen pools in the soil are unfortunately impossible. Thus we have to approximate the initial state of the soil.

We measured six stands near SMEAR II and five stands in Estonia about 400 kilometres south of our original stand. In each stand, we determined five size classes so that the biggest included 5%, the second 15%, the middle 30%, the second smallest 30% and the smallest 20% of the trees in the stand. Thereafter we took systematic samples of six trees from each size class. We measured retrospectively the annual diameters and heights until the age of three years. The identification of the youngest tree rings and whorls was problematic, especially in the smallest size class.

We used the tree characteristics five years after stand initiation as the initial state in the simulations. There was no information available describing the initial state of the soil in the stands. On the other hand, the fertility variation between the stands was clear when judged according to the size of the trees and according to ground vegetation. The fertility in some stands was rather similar to the stand around SMEAR II while some sites were rather poor. We assumed that the ratios between the soil macromolecules were everywhere the same as at SMEAR II. We further assumed that the pool of large carbon molecules varies between the sites determining the site fertility. In order to obtain a rough estimate of the pool of large macro molecules, we multiplied the initial soil carbon pool at SMEAR II with a site-specific constant. The value was one for sites that were as fertile as the site around SMEAR II and less than one for the sites of lower fertility. We determined the constant from the requirement that the diameter in the second biggest size class at the end of the simulation is equal to the actual measured diameter. We found that the fertility factor is one for the stand at Järvselja and 0.65 for the stand growing in Vihterpalu. Thus, the simulated pool of carbon macromolecules was considerably smaller in Vihterpalu, only 65% of that in the stand around SMEAR II whereas in Järvseljä, the pool equalled that of SMEARII.

### Comparison of the predicted and measured diameters and heights

The predictions were successful at the more fertile sites, both near SMEAR II and in Estonia. The predicted growth patterns on the poorer sites differed clearly from the measured ones so that the simulated trees grew too fast before canopy closure. We found that the simulated behaviour of ground vegetation underestimated the utilization of the nitrogen released in decomposition resulting in to high nitrogen availability to trees and too large growth. We re-estimated the values of the parameters describing the behaviour of the ground vegetation from the requirement that the available nitrogen content (nitrogen ions content) remains stable during the simulation also on poor sites. We used no additional measurements from the test stands in the redetermination, and we made a second round of predictions.

**Fig 4:**
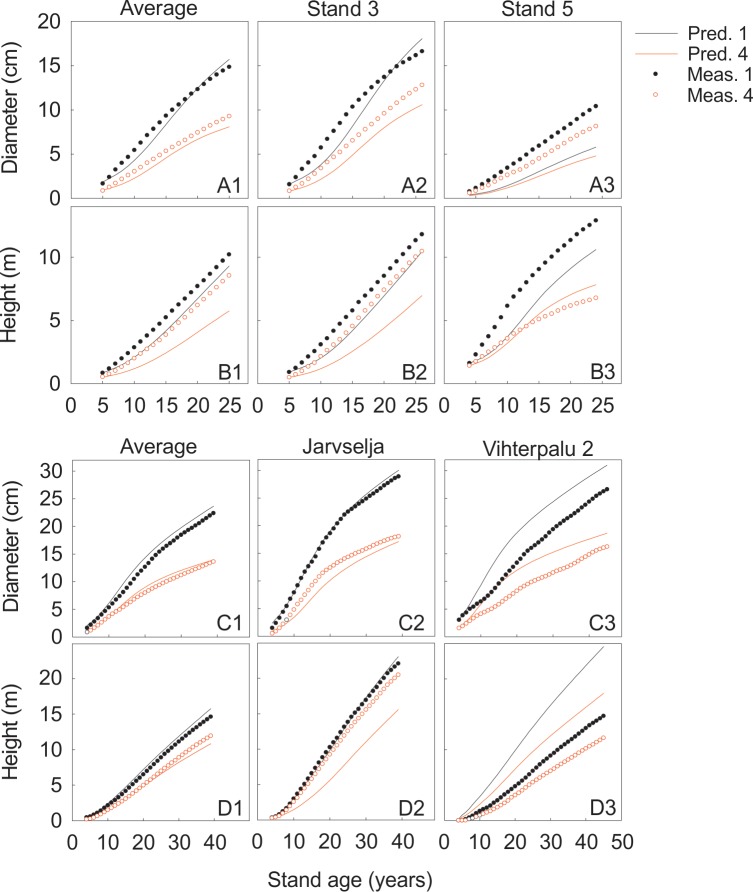
Predicted and measured height and diameter for first and fourth size classes. The first two rows (A–B) are from the stands near SMEAR II and the following ones (C–D) from the Estonian stands.The stand number 5, (*Vaccinium* type site, 4400 stems ha^–1^) had the poorest fit near SMEARII and stand number 3 (*Myrtillus* type site, 2000 stems ha^–1^) the best fit. In Estonia, Vihterpalu 2 had the poorest fit and Järvselja the best fit.

The second round of test predictions with field measurements, after the fine-tuning of the ground vegetation component, was successful both near SMEAR II and in Estonia (Fig 4). There was some discrepancy, especially in the smallest size classes, between the prediction and measurements of diameters and heights, but the means over the stands both in Finland and Estonia were very close to each other.

The residuals, i.e. the difference between the measured and predicted final diameters and heights revealed evident systematic behaviour as a function of the initial diameters indicating problems in determining the diameters of the small seedlings. We discovered no other systematic features in the residuals.

## Discussion

Our forest ecological theory is a result of theory development [28], field measurements and analysis of the obtained data with dynamic models during several decades. We started field measurements in the early 70s focusing on photosynthesis [11,29] and daily height increments [30,31]. The work expanded to the dynamic modelling of forest stands [32–36]. Material and energy fluxes gave a sound basis for theoretical thinking and for construction of measuring stations [27]. The research expanded to consider phenomena in the soil and in the ground vegetation [37,38]. The Newtonian approach to construct theories provided means to separate theoretical thinking and more technical dynamic modelling [11]. The summary of the research by our group is presented in the books by Hari and Kulmala [39] and Hari et al. [40].

We constructed our theory of forest ecosystems strictly according to the approach that Newton introduced in his book Principia Mathematica. In this way, we were able to express our forest ecological knowledge exactly and it resulted in testable predictions in the field. We think that the Newtonian approach to construct theories provided a very fruitful backbone to express our forest ecological knowledge gained during our research in an exact and operational form.

Our model NewtonForest includes two components, i.e. trees (vegetation) and soil. These two components differ strongly from each other. Trees have a powerful regulation system of formation of tree structure. The regulation system operates in the annual time scale. The annual tree rings and heights of whorls indicate the action of the regulation system. Thus the annual time scale is a very characteristic feature of Scots pines and we have to use the annual time scale to introduce properly the fundamental feature of Scots pine trees. We introduced the two time scales into NewtonForest to avoid discrepancy between the reality and applied model structures.

The mechanisms of the regulation are not understood, but we can use assumed operation principles of the regulation and combine the metabolism and regulation of tree structure in the modelling. We introduced two operation principles, i.e. balanced structure and efficient use of the available resources, and we obtained models to describe the development of tree structure.

Simultaneous use of two time scales is a characteristic feature in NewtonForest.

We analyse the development of soil with dynamic deterministic model using continual time. We introduce the metabolic mechanism with axioms that explain the behaviour of forest soil. In this way we can explain the soil development with known mechanistic metabolic phenomena [41].

The mathematical analysis of the forest ecosystem defined by the concepts and axioms was rather straightforwardly with numerical simulations. We started from the initial state with 5-year old trees approximately 1 m tall. We calculated the annual change in the state variables of the system and we obtained the state of the ecosystem one year later. We repeated this calculation of the annual changes and new states of the ecosystem over 50 years. In this way, we obtained predictions of tree diameter and height during the stand development.

The axioms are based on understanding of the most important processes in trees, ground vegetation and soil. This knowledge is not species specific and we can expect that the axioms are adequate also in other forest ecosystems. Thus evidently, we can apply the structure also to other ecosystems dominated by other tree species. The values of the parameters are surely species specific. Preliminary tests with Norway spruce have been encouraging.

In our simulations, we obtained the values of several parameters from the SMEAR II measuring station. The measuring station is planned and constructed to measure material and energy fluxes and amounts of organic material in the forest ecosystem around the station. Our theory deals with material and energy fluxes and amounts, and thus our theory and measuring station focus on the same aspects of the forest ecosystem. As a result, we needed to estimate the values of only 10 additional parameters, which we obtained from the observed behaviour of growths in the stand around SMEAR II.

The simulations begin from the initial state of the forest ecosystem some years after a stand-replacing disturbance, such as a clear-cut or a high-intensity forest fire. The determination of the initial state decades ago is quite problematic. Although coniferous trees store the history of the stand in easily measurable characteristics, such as tree ring widths and whorl heights. The difficulty in determining these characteristics during first years of the stand development caused difficulties in measurements, and limited our measurements to the time when the seedlings were taller than 1 m. Another problem was the initial state of the soil, since we have no retrospective measurements available.

We assumed that the rations of soil macromolecules are universal and not site specific. We approximated the soil initial state for each stand from the requirement that the simulated and measured diameters at the end of the simulation in the second biggest size class are equal. This procedure to determine the initial state of the soil introduced estimation bias into the fit between measured and predicted diameters in the second biggest size class.

Our analysis indicated big differences in sensitivities between the estimated parameters. NewtonForest was very sensitive to inaccuracies in the values of three parameters. The decomposition of proteins in the soil provides nitrogen for the vegetation and the nitrogen taken up by ground vegetation is essential for the concentration of plant available nitrogen after the establishment of the stand. These two parameters are connected with the nitrogen availability in the simulations. The third parameter deals with the sugars available for growth. NewtonForest is sensitive to the inaccuracies in the parameter values that affect the simulated resources for growth. Inaccuracies in the other simulated parameters have only minor effect on the simulated ecosystem development.

The predictions based on the mathematical analysis of the behaviour of the system defined by the concepts and axioms as well as their empirical tests play a very important role in the Newtonian theory development. We tested the predictions with quite extensive field measurements done near SMEAR II and in Estonia. The test of the predictions, were successful, especially in the three biggest size classes. However, there was some discrepancy in the predictions of diameters and heights in the smallest size classes. When we take the mean over the stands near SMEAR II and in Estonia, the predictions are very close to the observed ones. Evidently, inaccuracies in the initial states generate the discrepancy between simulations and measurements. Thus the predictions of the pattern and of the level of growth provided clear corroboration to our forest ecological theory. There seems to be strong regularities in the growth and development of Scots pine forest ecosystems and our theory is able to predict these regularities.

Statistical modelling of the development of forest stands has gained much interest in the last few decades. The statistical approaches are often based on large field measurements and on the relationships between tree growth and the dimensions of trees [5–7]. De Wit [42] started the carbon balance tradition in agriculture and this approach was also introduced into forest research [32]. According to our knowledge, there exists no other comprehensive forest ecological theory that is based on physiological knowledge and that simultaneously considers trees, ground vegetation and soil microbes. This limits the possibilities for comparing our approach with other forest ecological theories.

In conclusion, the Newtonian approach to construct and test theories proved to be very fruitful in forest ecology, as it provided a good formalism to proceed step-wise in the exact formulation of our ideas. We introduced new concepts to capture the most relevant features of Scots pine ecosystems, and we utilised physiological and structural knowledge to formulate the axioms. In addition, we quantified the axioms in exact forms that allowed simulation of the forest ecosystem behaviour and exact predictions of radial and height growth in five size classes of Scots pine trees. The predictions for six stands nearby SMEAR II and for five stands in Estonia were quite successful indicating that our theory of forest ecology is able to predict and explain the important regularities in the development of the Scots pine ecosystem.
